# Control of the Cu morphology on Ru-passivated and Ru-doped TaN surfaces – promoting growth of 2D conducting copper for CMOS interconnects†

**DOI:** 10.1039/d1sc04708f

**Published:** 2021-12-13

**Authors:** Cara-Lena Nies, Suresh Kondati Natarajan, Michael Nolan

**Affiliations:** Tyndall National Institute, University College Cork Lee Maltings, Dyke Parade Cork T12 R5CP Ireland michael.nolan@tyndall.ie; Synopsys Denmark ApS Fruebjergvej 3 2100 Copenhagen Denmark; NIBEC, School of Engineering, University of Ulster at Jordanstown BT37 0QB UK

## Abstract

Prolonging the lifetime of Cu as a level 1 and level 2 interconnect metal in future nanoelectronic devices is a significant challenge as device dimensions continue to shrink and device structures become more complex. At nanoscale dimensions Cu exhibits high resistivity which prevents its functioning as a conducting wire and prefers to form non-conducting 3D islands. Given that changing from Cu to an alternative metal is challenging, we are investigating new materials that combine properties of diffusion barriers and seed liners to reduce the thickness of this layer and to promote successful electroplating of Cu to facilitate the coating of high-aspect ratio interconnect vias and to allow for optimal electrical conductance. In this study we propose new combined barrier/liner materials based on modifying the surface layer of the TaN barrier through Ru incorporation. Simulating a model Cu_29_ structure at 0 K and through finite temperature *ab initio* molecular dynamics on these surfaces allows us to demonstrate how the Ru content can control copper wetting, adhesion and thermal stability properties. Activation energies for atom migrations onto a nucleating copper island allow insight into the growth mechanism of a Cu thin-film. Using this understanding allows us to tailor the Ru content on TaN to control the final morphology of the Cu film. These Ru-modified TaN films can be deposited by atomic layer deposition, allowing for fine control over the film thickness and composition.

## Abstract 2

Modifying the surface layer of the barrier material TaN with Ru controls the morphology of deposited copper.

## Graphical abstract

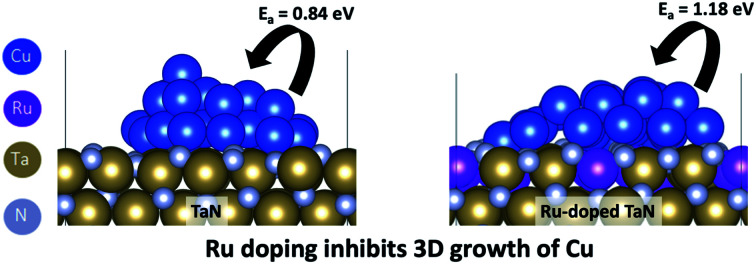

## 1. Introduction

**Fig. 1:**
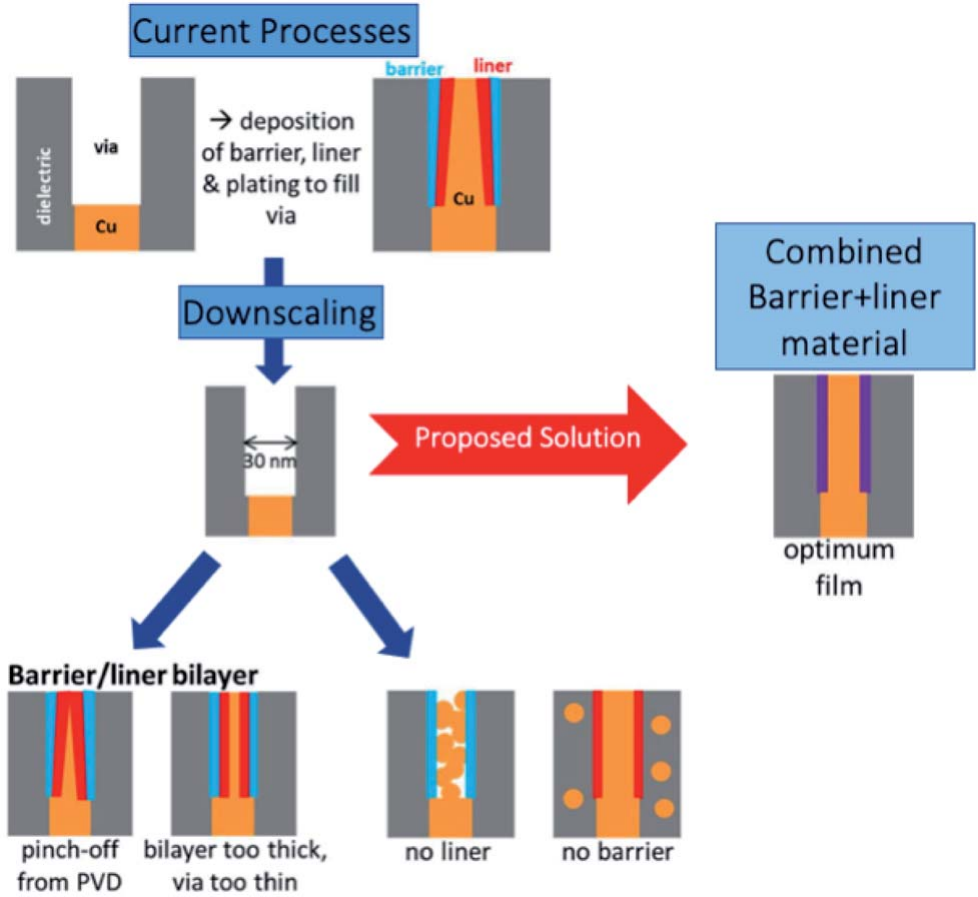
Overview of issues related to downscaling of Cu interconnects and a proposed solution, adapted from Natarajan *et al.*^9^

The size of transistors in electronic devices is steadily decreasing, keeping Moore's law on track into the 2020s. However, continued advancements in this scaling are becoming limited by the scalability of the lowest level device interconnects, which will create a severe bottleneck for future nanoelectronic device miniaturisation.^1,2^ These interconnects are currently made of copper metal, which needs to be deposited inside a via which contains a barrier material, such as TaN, the function of which is to prevent diffusion of Cu into the dielectric. Also present is a liner material (which is also referred to as a seed layer, glue layer or adhesion promoter) that facilitates the electroplating of a smooth Cu thin film, Fig. 1.^3–6^ Otherwise copper tends to form non-conducting islands.^3,7,8^

However, as the dimensions of transistor devices decrease, this setup becomes problematic. For example, difficulties arise in depositing two additional layers of the material and the Cu interconnect in the high aspect ratio interconnect via.^10^ Further, Cu exhibits significantly increased resistivity at such small scales^11,12^ as a result of forming non-conducting islands rather than smooth conducting films. The narrowness of the via also makes traditional physical vapour deposition less attractive since there will be a pinch-off effect at the top of the via, as shown in Fig. 1.

Taken together, these factors clearly necessitate new developments in materials for interconnects. We propose that a single material which is designed to combine the barrier and liner properties required for Cu deposition can be developed and demonstrated. This material is composed of a barrier material with incorporation of another metal species in the terminal layer that promotes copper wetting and hence conducting film deposition. Such a material will eliminate the extra seed layer, taking up a reduced volume in the via and reducing the number of processing steps. This will permit a larger volume for copper deposition thus maintaining the low resistivity required for interconnects. Such a combined material will be most favourably deposited using atomic layer deposition (ALD), which has no challenges in depositing conformal, thin and uniform films on a range of substrates. ALD can deposit this film simply by switching the metal precursor from that of the barrier layer to that of the second metal in the ALD cycle.

There has been intense interest in the search for materials that function as good barriers and liner layers and many different materials have been studied as diffusion barriers or liner materials, while there are fewer cases of combined diffusion and liner materials. There has been a significant level of experimental work on different diffusion barrier materials since they were first used when the industry changed from Al to Cu interconnects in 1997.^13–24^ These barrier materials can be divided into several different categories, according to the classification of Kaloyeros and Eisenbraun.^25^ Of these, refractory metals, *e.g.* Ta, W, Ru, and secondary or tertiary materials of these metals are the most successful. This is due to the high melting points of refractory metals, which has the advantage of limiting grain boundary diffusion at operating temperatures, one of the main failure mechanisms in Cu interconnects.^3^

Even though Gall^26^ found that to maintain resistivity at a minimum, an interconnect metal that does not need a barrier or a liner material would be ideal, in order to extend Cu interconnects beyond current technology nodes and to keep the material cost low, a combined barrier + liner material is of the essence. In this area, studies of potential liner materials are carried out in conjunction with a barrier material, either known or novel, or in testing of a potential combined barrier and liner material.^7,15,16,27–34^ In experimental work, the primary characteristics of a potential liner for Cu deposition include adhesion of Cu to the liner,^15,29,32,34^ wettability of Cu,^7,15,30,32^ resistivity^27–32^ and electromigration reliability.^31,33^ Adhesion is studied through a simple tape exfoliation test: if the metal and liner remain adhered, then the adhesion is deemed strong enough under typical manufacturing conditions. Wettability can be confirmed through study of the uniformity of the interconnect metal using STEM, TEM or SEM. Resistivity can be measured directly by passing a current through the device. Lastly, electromigration and reliability tests are carried out by studying the time to break down, and through observation of any structural changes as seen through STEM. Typically, voids will be formed at one end of the interconnect, while atoms agglomerate at the opposite end. The performance of a barrier material is generally assessed through studying interfaces and microstructures formed between the barrier, Si and Cu after annealing at different temperatures.^13,17,20,21,23,24^ Other approaches include determining if Cu can be plated in a continuous fashion, which is often done in conjunction with testing a liner material.^15,16,22^

In an important study Han *et al.*^4^ used first principles density functional theory (DFT) simulations to develop criteria for determining if a candidate material will act as a suitable liner layer between an interconnect metal and a barrier material based on the computed adhesion energies of a monolayer of Cu on TaN (111) and on TaN (111) that is decorated with a monolayer of the potential liner material. They propose that for a given interconnect and diffusion barrier, an appropriate liner material can be determined through comparison of the relative adhesion energies between the three materials. The following criteria were developed:

(1) If the adhesion of the interconnect metal on the liner material is stronger than that on the diffusion barrier, agglomeration of the interconnect will be prevented.

(2) Agglomeration of the liner material and diffusion into the interconnect metal can be prevented if liner adhesion to the diffusion barrier exceeds the adhesion of the interconnect to both the diffusion barrier and the liner material.

They found that in the Cu/liner/TaN system these criteria were met when using Ru, Nb, Zr and Ti as the liner, while Co, Al and Ni did not satisfy the conditions. These results were further confirmed through *ab initio* molecular dynamics (AIMD) calculations at a typical process temperature of 500 K and comparison to the existing experimental results.^16,28,35–37^ However, the focus of that work was solely on the adhesion energies in the Cu/liner/TaN stack and did not take into account the many different energies that contribute to the growth process. It does not give any insights into controlling the morphology of copper on the liner/barrier material. Understanding the growth mechanism in more detail allows us to choose material combinations that will prevent agglomeration in a more targeted way.^4^

Using first principles simulations, *e.g.* with density functional theory (DFT), we can determine if a candidate is a potentially useful barrier material by studying the energy barriers for Cu to migrate through the material.^38,39^ DFT simulations show that the replacement of surface Ta in TaN with a Cu atom is less favourable than adsorption of Cu on TaN by *ca.* 2 eV, indicating that the barrier to Cu migration is at least 2 eV and that migration of Cu into the barrier will require a significant amount of energy. This confirms that our results match experimental knowledge of using TaN as a diffusion barrier. Therefore, we can use TaN as the barrier layer and assess options for a second metal that can induce the required liner properties. In our work, we aim to use first principles simulations to discover single materials that can combine the properties of the barrier and liner to prevent Cu diffusion and promote deposition of conducting Cu. Based on the literature discussed above, the most important factors in selecting a suitable barrier and liner material, and therefore any combined barrier + liner material, are:

● Thermal stability

● Low grain boundary diffusion

● Low overall resistivity

● Wettability and adhesion of Cu

● Low electromigration

Since not all of these can be studied using DFT and will therefore need experimental work to validate, we have previously studied the adhesion and migration of Cu on Ru-modified TaN and demonstrated that one and two monolayers of Ru on TaN comprise a suitable liner/barrier stack for the Cu/TaN system. We also examined the incorporation of Ru through Ru substitution on Ta sites in the terminal layer of TaN to produce a single liner/barrier material. We found that the presence of Ru in the system enhances the overall strength with which Cu binds to TaN. Further, Ru dopants in the TaN surface act as a nucleation point on the surface, with single Cu atoms preferentially migrating towards these sites. However, as the concentration of Ru doping increases, the mismatch in the atomic radius between Ta and Ru causes surface distortion and recess formation. These recesses can trap Cu atoms and enhance the strength of Cu–surface interactions.^9,39^ As the first criterion to establish the wettability of Cu, we use the Cu binding energy compared to the cohesive energy of bulk Cu as a reference. If the strength of Cu binding to the surface exceeds the cohesive energy of bulk Cu, we predict that wetting will be favourable as the film grows. We find for small Cu clusters (with up to 4 Cu atoms) that this condition is met by a Ru-doped TaN surface in which all Ta atoms in the terminal surface layer are replaced by Ru, denoted as Ru^100^, and a 1 monolayer (ML) Ru-passivated surface.

In this study, we focus on the competition between copper wetting and island formation on Ru-modified TaN, using a model structure for copper that has 29 atoms, as used in ref. 40. This model is substantially larger than our previous models and allows us to study in detail the interactions that control the growth of copper, namely Cu–Cu and Cu–surface interactions, as well as transitions from 2D to distinct layered structures and associated activation barriers to copper migration. Overall, this model gives a more realistic representation of 2D *vs.* 3D growth than can be achieved with smaller copper cluster models but also advances the work in ref. 4 by explicitly demonstrating the factors that control the morphology of copper deposited on a barrier + liner layer.

To understand wettability in more detail, it is important to go beyond simply determining adhesion properties^4^ and deliver insights into the role of the composition of the combined barrier/liner materials in the growth mechanisms of copper films. Particularly important in this regard are the activation barriers for metal migration from the substrate onto a nucleating island which will determine if the island continues to grow into a 3D structure or will wet the surface.^41–43^

In the growth of thin metal films, there are typically two dominating mechanisms which depend on the strength of the interaction with the substrate. Films grown on strongly interacting substrates, *e.g.* silver on silver substrates, generally follow classical homoepitaxial growth, where a higher temperature is associated with 2D growth of the thin film, allowing annealing as a facile approach to ensure a smooth layer. On weakly interacting substrates, where the interaction between the atoms in the metal film is stronger than the interaction with the substrate, the growth mechanism changes to 3D, leading to rough surfaces and faceted nanostructures instead of the smooth film desirable for interconnects.^41–47^ This allows us to conclude that by varying the strength of the interaction between the film and the substrate but additionally modifying the activation barrier for metal migration, in our case through doping and passivation of TaN with Ru, we can control which growth mechanism will be promoted and thus the final structure of deposited copper, although some differences to a classical homoepitaxial mechanism can be expected, as this system is not homogenous.

It is difficult to unambiguously define what constitutes a strongly or weakly interacting substrate. Gervilla^48^ defines a weak interaction, when the bond strength between the metal and the substrate is at least half the bond strength of the metal, based on a review of thin film growth on oxides by Campbell.^49^ For this work we will consider the adsorption energy compared to the cohesive energy of Cu as a guide to the strength of the interaction. Here, if the binding energy is close to or greater than the cohesive energy of the metal, the interaction will be considered strong.

To the best of our knowledge, work on modelling the growth mechanisms of these kinds of systems is typically carried out using kinetic Monte Carlo (kMC) or mean field approximation methods,^41–43,47^ although some work is carried out using classical molecular dynamics.^44,46^ Using these methods, Gervilla *et al.* were able to show that higher temperatures can promote top-layer nucleation which promotes island formation, but that this is also controlled by the type of side wall facet.^41^ Further, Grillo *et al.* were able to show that in the Ru atomic layer deposition (ALD) process, the size of the nanoparticle cannot be controlled through the number of ALD cycles. Instead, to control the nanoparticle size and morphology, the process must be carried out at temperatures below 100 °C, where atom attachment drives the growth, rather than nanoparticle diffusion and coalescence.^42^ Meanwhile, in two separate studies, Jamnig *et al.* showed that the size at which metal clusters remain mobile depends on the metal used^44^ and that the introduction of a gaseous surfactant such as N_2_ at different stages of the growth can promote either 2D or 3D growth.^45^

Through the combination of several computational techniques along with the understanding of the types of growth mechanisms that occur depending on the interactions at the interface between the metal and substrate, we are able to predict that 3D growth of Cu is inhibited on TaN surfaces with 1 ML Ru passivation or 50% Ru doping. Passivation of TaN with 1 ML does not change the number of process steps, and it can be challenging to deposit 1 ML of a metal. Thus, the Ru-doped TaN surface will reduce the number of processing steps and in addition displays more properties required for a combined barrier/liner material.

## 2. Methods

All calculations in this study were carried out using the Vienna *Ab initio* Simulation Package (VASP5.4).^50^ This is based on periodic, spin polarised density functional theory. We use the Perdew–Burke–Ernzerhof (PBE) approximation to the exchange–correlation functional.^51^

The valence electrons are expanded in a periodic plane wave basis set using a kinetic energy cut-off of 400 eV. The valence electron configurations used for Ta, N, Ru and Cu are as follows. Ta: 6s^2^5d^3^; N: 2s^2^2p^3^; Ru: 5s^1^4d^7^; Cu: 4s^1^3d^10^. The core-valence electron interactions are treated by the projector augmented wave (PAW) potentials.^52^

The descriptions of bulk epsilon-TaN, the low energy (1 1 0) surface, and Ru doped and passivated TaN have been published previously and the VASP files are available on https://github.com/MMD-group/VASP.^9,39^ A summary of the details of these models can be found in the ESI.† To accommodate Cu_29_ structures we use the (2 × 4) TaN supercell described in ref. 39 with the following dimensions: *a* = 18.11 Å, *b* = 23.36 Å, and *c* = 30.48 Å; *α* = *β* = *γ* = 90°. We find that all of the TaN surfaces studied are metallic. To prepare our combined liner/barrier system, we doped Ru onto Ta sites in the top layer of the surface and not throughout the slab; such a composition can be achieved using atomic layer deposition of TaN and then using a Ru precursor instead of the Ta precursor for the top layer.

The first study of doping was carried out with a single Ru dopant in the surface.^39^ Replacing either a three-coordinate (F-site) or six-coordinate (S-site) Ta atom with a Ru atom, showed that doping is more favourable at the F-site. For our second study,^9^ we explored doping concentrations of 25%, 50%, 75% and 100% Ru in the surface layer. Using 50% Ru doping as an example, the effect of the distribution of Ru in the surface was studied. We found that the distribution of dopants changes the stability, which is primarily driven by the ratio of S to F site doping. Surfaces with more F site doping are more favourable. Furthermore, as a result of the smaller Ru ionic radius compared to Ta, recesses are formed in the surface that can trap Cu atoms, and the size and density of these are dependent on the distribution and concentration of Ru dopants.

Comparing 1 ML and 2 ML Ru passivation of TaN (110) showed that 2 ML of Ru behaves similarly to bulk Ru. Promising properties of a barrier + liner material were found for 1 ML of Ru deposited onto TaN. While this is not a single material, a monolayer of Ru would certainly free up some volume within the via for Cu deposition.

We use eqn (1)–(4) to determine the energetic quantities of interest for this work. The binding energies of copper on the modified TaN surfaces are calculated from eqn (1), where we use an isolated, free copper atom as a reference:1
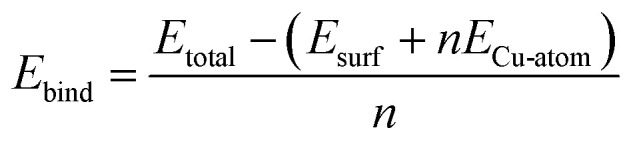
*E*_total_ is the total energy of the relaxed system with adsorbed Cu atoms and *n* is the number of Cu atoms. *E*_surf_ is the single point energy of the TaN surface after Cu adsorption and with the Cu adatoms removed. Similar to ref. 9, we use this as a reference energy for Ru-doped or Ru-passivated TaN instead of the energy of the bare surface before Cu adsorption, as we found that surface rearrangements can occur during the relaxation with adsorbed copper, usually resulting in a surface with a lower energy. Using the energy of the modified TaN surface produced after Cu adsorption as a reference therefore eliminates any bias in the binding energy caused by the energy required for this surface rearrangement. Given the magnitude of the binding energies, we did not include van der Waals interactions.

In eqn (2) we compute *E*_bind*_ which is similar to *E*_bind_ but uses the gas phase Cu_*n*_ cluster as a reference.2
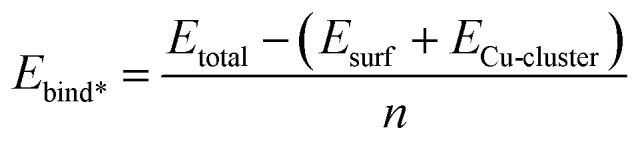
where *E*_total_, *n* and *E*_surf_ are the same as in eqn (1), and *E*_Cu-cluster_ is the single point energy of the adsorbed Cu_*n*_ cluster with the surface removed. This gives us an indication of the magnitude of the metal–surface interaction and is used as a reference only.

Using *E*_bind_ and *E*_bind*_ together we can isolate the Cu–Cu interaction energy using eqn (3):^9^

3 *E*_Cu–Cu_ = *E*_bind_ − *E*_bind*_

As discussed in ref. 41–47, the competition between the metal–substrate interaction and the metal–metal interaction is a key feature in the thin film growth mechanism. A strong metal–substrate interaction (with *E*_bind_ ≥ *E*_cohesive_), and a metal–substrate interaction that exceeds the metal–metal interaction are factors that contribute to promoting thin film wetting.

We relaxed the atomic structures of Cu on Ru-TaN with different Ru contents at 0 K in static DFT relaxations, using Monkhorst–Pack Γ-point sampling, Gaussian smearing, with *σ* = 0.1 eV and convergence criteria of 1 × 10^−4^ eV in the energy minimization and 0.02 eV Å^−1^ as the force threshold in the ionic relaxations. We also ran *ab initio* MD simulations in the *NVT* ensemble (constant number of particles, *N*, constant volume, *V*, and constant temperature, *T*) with the following inputs: the kinetic energy cutoff is 250 eV; temperature is 300, 500 and 800 K. A time step of 5 fs over a total of 1000 steps was applied to each run, yielding a simulation of 5 ps in length. The atoms in bottom two layers of the TaN surface slab are frozen. The Cu_29_ structures optimised at 0 K on various Ru-TaN surfaces are used as the starting geometries for the AIMD calculations. These AIMD simulations are used to explore how Cu atoms may migrate to form Cu islands on our set of Ru-TaN materials. Movie files (animated gifs) of AIMD runs at 500 K for TaN, 1 ML-Ru on TaN, 50% Ru-TaN and 100% Ru-TaN have been provided.

To characterise the impact of the TaN modification on the structure of adsorbed copper, we examined the position of the Cu atoms during the 0 K relaxation and during the finite temperature MD simulations as a function of MD time. We collected the *z*-coordinate (coordinate normal to the surface) of each Cu atom at each relaxation or time step, from the VASP XDATCAR file. The *z*-coordinates are shifted such that the original position of the copper atom is taken as zero and we can plot the resulting change in the *z*-coordinate with respect to the relaxation or time step.

4 Δ*Z* = *Z*_*n*_ − *Z*_0_

Here, *Z*_*n*_ is the *z*-coordinate at the *n*^th^ relaxation/time step *n* and *Z*_0_ is the *z*-coordinate at the first step. This yields information about which atoms are displaced from their original positions in the adsorbed 2D copper structure and migrate to form a second or third layer in the Cu_*n*_ structure or if copper atoms migrate into the surface layer. Our convention is that copper atoms migrate into the next layer if they are displaced along the *Z*-direction by more than 1 Å from their original position. For migration into the TaN surface, an atom is buried, when it is displaced by more than −1 Å (downwards) from its original position.

Activation energies for migration of atoms between copper layers were calculated using the climbing image nudged elastic band (CI-NEB) approach^53,54^ with five images and with the following equation:

5 *E*_A–B_ = |(*E*_A_ − (*E*_TSAB_))|

Here, *E*_A–B_ is the activation energy for migration of an atom from state A to state B, *E*_A_ is the total energy of state A and *E*_TSAB_ is the total energy of the transition state between state A and state B. Total energies were converged to 10^−4^ eV and forces to 0.03 eV Å^−1^

Electronic charges were computed using the Bader charge partitioning scheme.^55,56^ All geometry images were created using the “Visualization for Electronic and Structural Analysis” (VESTA) package.^57^

## 3. Results & discussion

In this work, our model system is a 29 atom copper structure, that is interfaced with Ru-TaN; this cluster has been used in DFT studies of copper–MoS_2_ chemistry^40^ and is large enough to show distinct 2D and 3D structures and permit analysis of Cu migration, while still being computationally tractable. All calculations begin with the 2D structure of Cu_29_ adsorbed on the various Ru-modified TaN surfaces and we explore how the structure evolves during 0 K relaxations and MD simulations at finite temperatures. Ru doped TaN surfaces will be henceforth referred to by Ru^*x*^, where *x* is the doping concentration. Density of states and Bader charge analysis are presented in the ESI.†

**Fig. 2:**
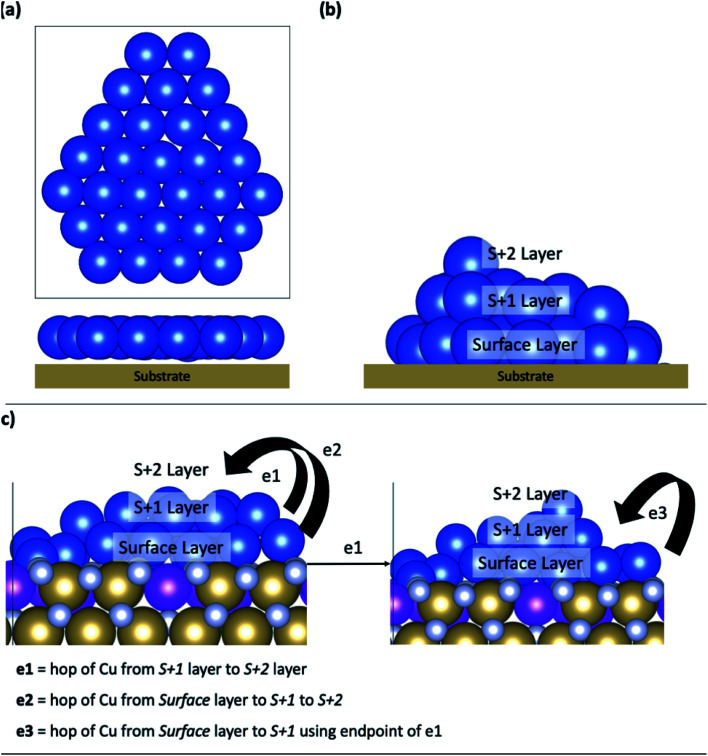
(a) The Cu_29_ model adsorbed and relaxed on all surfaces and (b) labels used to refer to the different layers of 3D structures. (c) Schematic describing different copper atom transitions between different layers in copper interfaced with the Ru-TaN surface. Cu is shown in blue, Ru in purple, Ta in gold and N in silver for all figures.

Fig. 2(a) shows a schematic structure of Cu_29_ adsorbed on a substrate with a 2D structure. 0 K relaxation calculations were carried out on each TaN and Ru-TaN surface. For each relaxed structure three MD calculations were performed for 5 ps at 300 K, 500 K and 800 K (each starting from the 0 K relaxed structure). From analysis of these structures, selected nudged elastic band calculations were further carried out to determine the activation energy for the transition of copper atoms between layers, which are labelled in Fig. 2(b).

Potential Cu atom transitions are shown in Fig. 2(c) and include Cu atom migration from the layer directly bound to the surface, the surface layer, to the first layer away from the surface, the S + 1 layer or from the S + 1 layer to the next layer away from the surface, the S + 2 layer. We also consider Cu migration from the surface layer to the S + 1 layer after a Cu atom has migrated to the S + 1 layer. This is motivated by the findings that upward migration of a metal atom from the surface layer can promote the further migration of metal atoms from the surface, thus enhancing island formation.^47^

### Structure of copper on bare TaN and 1 monolayer Ru passivated TaN

**Fig. 3:**
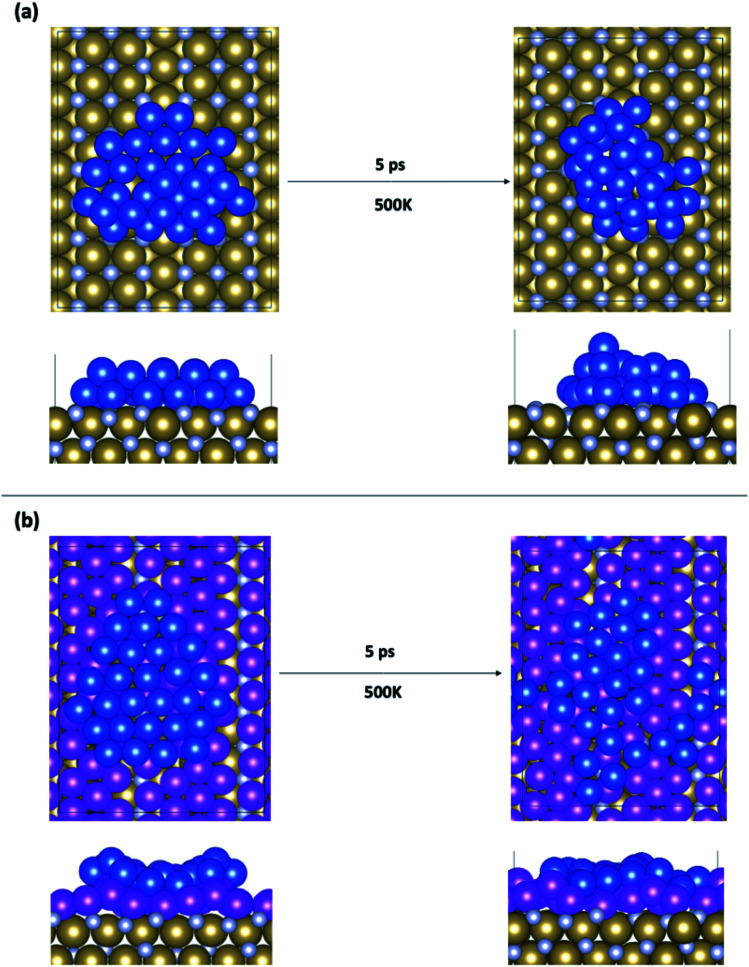
Structures of Cu_29_ obtained after relaxation and 500 K MD on (a) bare TaN and (b) 1 ML Ru passivated TaN.

We begin the analysis with the structure of two extreme examples. Adsorption of copper on the bare TaN surfaces is shown in Fig. 3(a), and on 1 ML Ru-passivated TaN is shown in Fig. 3(b); in both figures we show top and side views after relaxation and the final snapshots after 5 ps MD runs at 500 K. In the ESI, Fig. S3† we show snapshots for the other MD runs at 300 K and 800 K.

The binding energies of the relaxed Cu_29_ structures are shown in Table 1. The binding energy of Cu_29_ on bare TaN is −3.49 eV per atom, which is the least favourable binding energy of all the surfaces studied. The structure of Cu on the Ru^25^ and Ru^50^ surfaces is discussed in the next section. The 1 ML Ru-passivated surface has the most favourable *E*_bind_, with −4.12 eV/Cu. Note that a high Ru content increases the binding energy of copper on Ru-modified TaN.

**Table 1:** Binding energies and Cu–Cu interaction energies of the Cu_29_ cluster on each Ru-modified TaN surface. Ru^*x*^ indicates the Ru doping content in the TaN surface layer

Surface	*E*_bind_ (eV per atom)	*E*_bind*_ (eV per atom)	*E*_Cu–Cu_ (eV per atom)
TaN	−3.49	−0.74	−2.75
Ru^25^	−3.58	−0.85	−2.72
Ru^50^	−3.59	−0.85	−2.74
Ru^100^	−3.64	−1.04	−2.59
1 ML Ru passivation	−4.12	−1.46	−2.66

On bare TaN we observed a spontaneous transition from the initial 2D structure to a two-layer structure during the 0 K relaxation, as shown in Fig. 3(a), indicating that the TaN layer is not able to promote 2D wetting of copper; this is consistent with the known properties of TaN. In the finite temperature MD simulations, we see that further upward copper migration takes place. Copper atoms migrate from the S + 1 layer to form a third layer (S + 2) and we see an incipient (111)-like facet on the copper island. Bare TaN therefore promotes the formation of 3D copper islands. The TaN surface itself remains unaffected during this relaxation and no Cu atoms migrate into the surface, as expected, given the known barrier properties of TaN.

Next, we consider how our copper model interfaces with 1 ML Ru passivation of TaN. From our previous studies on adsorbates with 1–4 copper atoms,^9,39^ we know that Ru-passivation increases the strength of binding of Cu compared to Cu binding on TaN and that Cu atoms prefer to remain separated rather than associating. This is confirmed during the relaxation of 2D Cu_29_, in which there is no migration of copper from the surface layer to the S + 1 layer and copper remains flat while rearranging to conform to the corrugated Ru-passivated surface. This indicates that 1 ML Ru can indeed promote copper wetting.

During the MD calculations the flat structure persists at temperatures up to 800 K (which exceeds process temperatures during back end of line processing), and we observe some of the copper atoms at the edge of the sheet migrating away from the remainder of the cluster. This shows that association of copper, which is required for the formation of the 3D structure, is not favourable at finite temperature on the 1 ML Ru-passivated surface. This originates in part from the strong Cu–surface interaction (Table 1) facilitated by the incorporation of some Cu atoms into the Ru layer, which inhibits 3D migrations.^9^ Fig. S4 in the ESI† shows the change in the *z*-position of Cu_29_ on Ru passivated TaN to illustrate the incorporation of Cu into the Ru layer. This shows that, depending on the temperature, up to 4 Cu atoms migrate downwards by up to 2 Å, which means they have migrated into the Ru layer. This has no impact on the metallic character of the Ru monolayer or copper. There is also no migration of copper into the TaN layer, confirming that the barrier properties are unaffected.

If we compare the computed Cu–Cu interaction energies (see Table 1), then we see that they are weaker on the 1 ML Ru-TaN surface compared to the bare TaN surface and are the weakest for the highest Ru contents. This is consistent with our observation that copper atoms prefer to separate on the Ru-passivated TaN surface. This analysis shows that the presence of Ru on the TaN surface enhances the Cu–surface interaction. This is, in part, due to the stronger interaction between Cu and Ru compared to Cu and Ta.^39^ Further, we find that the Cu–Cu interaction energy is the strongest on the bare TaN surface. This also partially explains why Cu atoms prefer to associate on this surface.

Overall, these findings indicate that a monolayer of Ru deposited on TaN is effective as a liner for Cu. Manufacturing interconnects using Ru-passivated TaN will significantly thin the barrier/liner stack; however a single material is preferable as it can be thinned further and deposited in a single deposition run.

### Impact of Ru incorporation in TaN on 2D/3D copper stability

In this section we examine the impact of Ru doping in the surface layer of TaN to develop a single barrier + liner material. To avoid disruption of the barrier function of TaN caused by the difference in the atomic radius between Ta and Ru and the lattice mismatch between Ru metal and TaN, we chose to incorporate Ru dopants only into the surface layer of TaN, rather than throughout the material.

This type of film can be grown using ALD,^29,58,59^ which is ideal for high aspect ratio structures such as interconnect vias and controlled incorporation of a second cation into the barrier film.

**Fig. 4:**
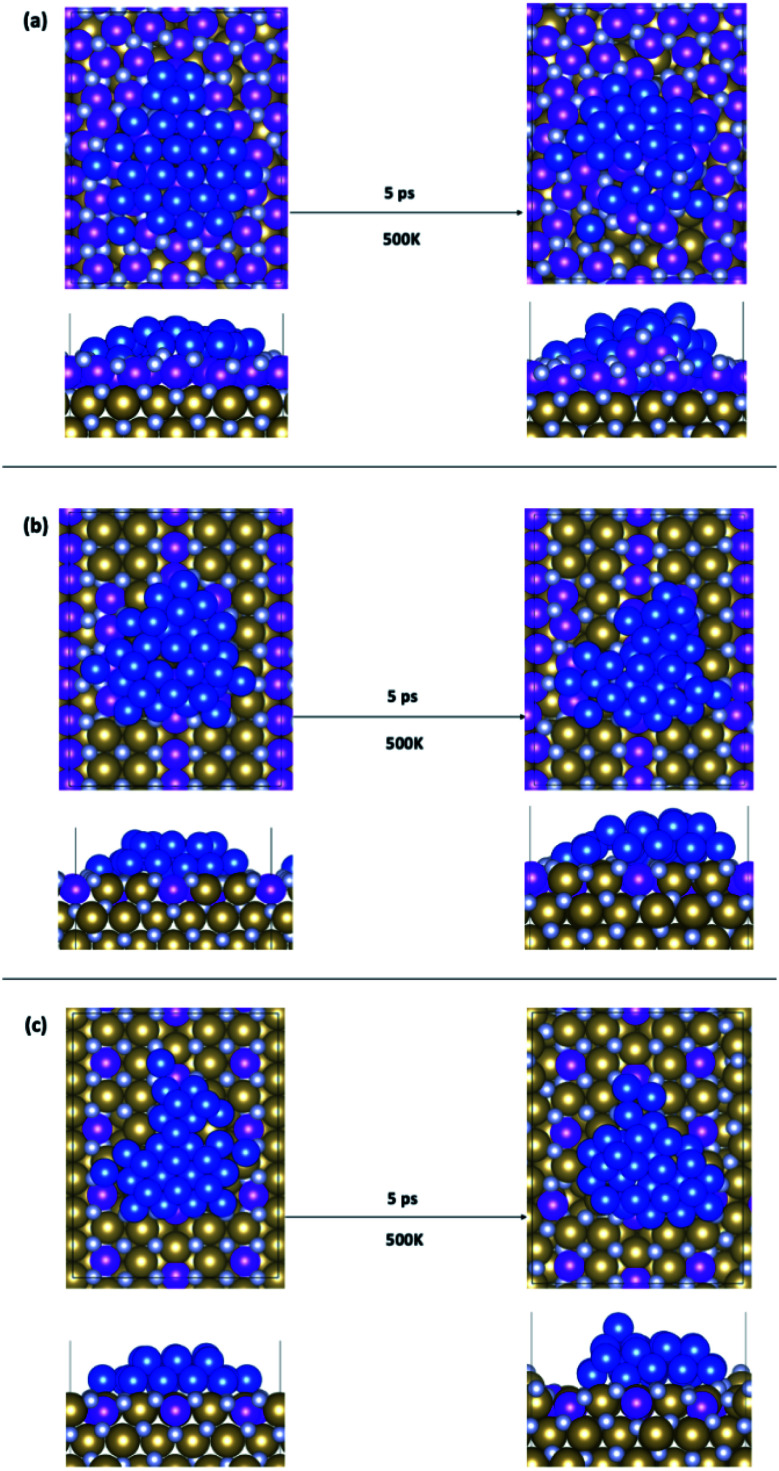
Structures of Cu_29_ obtained after relaxation and 500 K MD on (a) Ru^100^, (b) Ru^50^, and (c) Ru^25^.

**Fig. 5:**
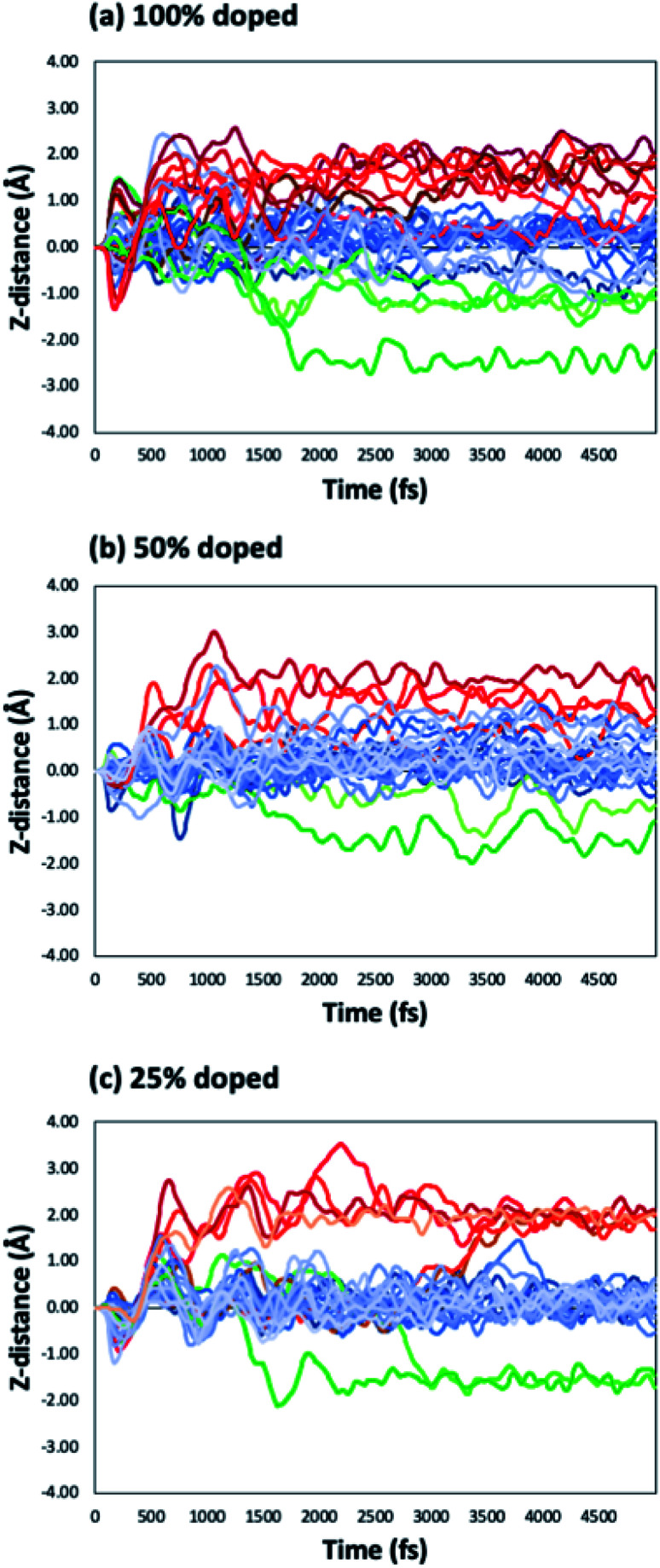
Change in the *Z*-position with respect to MD simulation time for Cu_29_ on (a) Ru^100^, (b) Ru^50^ and (c) Ru^25^ during an MD run at 500 K.

We have considered three examples of Ru incorporation, with 100%, 50% and 25% substitution of Ta with Ru. Top and side views of Cu_29_ after relaxation and after a 5 ps MD run on Ru^100^, Ru^50^ and Ru^25^ at 500 K are shown in Fig. 4(a), (b) and (c), respectively. Fig. 5(a)–(c) show the corresponding plots of the change in the *Z*-coordinate of the Cu atoms during the 500 K AIMD simulation. The results of 300 K and 800 K AIMD calculations for each Ru content are shown in ESI, Fig. S3.† Plots of the change in the *Z*-coordinate at all temperatures for Ru^100^, Ru^50^ and Ru^25^ are shown in Fig. S5 to S8.†

The Ru^100^ surface, has the second most favourable *E*_bind_ out of the surfaces studied, with *E*_bind_ = −3.64 eV/Cu, Table 1. Examining the plots of the *z*-coordinate *vs.* the relaxation step in Fig. 5(a), we see that during the relaxation four copper atoms migrated up to 1.7 Å into the Ru-TaN surface. As 3 Cu atoms appeared to migrate out of the 2D structure, this upward migration is <1 Å, and overall, we can say that the copper film is not transitioning to a 3D structure, as can be seen in the top view shown in Fig. 4(a). Also, we note some Ru migration upwards into Cu after the MD run is completed. This may be promoted by the highly distorted surface layer in Ru^100^.^9^

Migration into the surface is facilitated by surface recesses that are formed as a result of Ru doping, due to the smaller atomic radius of Ru compared to TaN.^9^ These form on all Ru-doped TaN surfaces studied. The size and density of recesses formed depends on the percentage of Ru and the distribution of the Ru atoms in the surface, as discussed in ref. 9. This distortion of the surface structure facilitates the movement of Cu atoms into the Ru-TaN layer. At 500 K Cu atoms begin to exchange with surface Ru and N atoms, creating a mixture of Cu, Ru and N and individual layers are no longer distinguishable in the structure. Fig. 5(a) shows the migration of Cu atoms both upwards and downwards into the surface, by as much as 3 Å from their original positions. However, as the deposited copper structure now contains both Ru and N, it is not immediately obvious if such a thin film would be sufficiently conductive or thermally stable for use in interconnects. Certainly a Cu–Ru intermetallic layer would be metallic, however nitrides of copper and ruthenium can be semiconducting, semi-metallic or metallic, depending on the exact composition and phase.^60–62^ Despite this, as we have noted in our previous work,^9^ atoms that migrate into the Ru-TaN surface layer do not migrate any deeper into the TaN film, thus confirming that the presence of Ru in the surface layer does not negatively impact the barrier properties of TaN.

For 50% Ru doping, we focused on what we termed the Ru^50-10^ surface,^9^ which is the most stable of the ten different dopant distributions studied for 50% surface doping.^9^ This is referred to as Ru^50^ throughout the main text; examples for the remaining Ru^50^ surfaces are discussed in the ESI.† The binding energy of Cu_29_ calculated on this Ru^50^ surface is −3.59 eV/Cu. The single layer copper transitions into a two-layer structure during the relaxation. The plot of copper *z*-coordinates presented in Fig. 5(b) shows that two atoms have been incorporated into the surface layer and three atoms have transitioned slightly upward from their original positions during the 500 K MD calculations. Study of the geometry shown in Fig. 4(b) shows that this upward migration is not sufficient to confirm the formation of the S + 2 layer and therefore we conclude that 50% Ru doping prevents formation of 3D copper islands.

On the Ru^25^ surface, Cu_29_ binds with similar strength to the bare TaN and Ru^50^ surface, with *E*_bind_ = −3.58 eV/Cu. We observe during the relaxation a spontaneous transition from the single layer to a two-layer structure, similar to what is observed for the bare and Ru^50^ surface (see Fig. 4(c)). In a 300 K MD run, the two-layer structure of copper persists. At 500 K, we observe that over the short simulation timescale a single atom migrates from the second to the third layer. Over longer timescales, beyond those accessible to our simulations, this migration will promote further upward Cu migration to produce a 3D morphology. The split between layers is very clearly visible in the plot shown in Fig. 5(c), where there is very little fluctuation in the *z*-position once atoms have moved either into a surface recess or a new layer.

Some of the behaviours of Cu atoms on surfaces with different Ru contents can be explained through study of the competition between Cu–Cu and Cu–surface interactions. As the Ru content increases the contribution of Cu–Cu interactions to the overall binding energy decreases and the Cu–surface (*E*_bind*_) contribution increases. The increased Cu–surface interaction causes the promotion of 2D growth. However, both energies must be considered together, as the Ru^100^ surface has the least favourable Cu–Cu interaction energy of −2.59 eV/Cu, but the Cu_29_ structure does not remain completely flat, as the competing Cu–surface interaction of −1.04 eV/Cu is not sufficient to prevent overall 3D growth.

This does not explain, however, why Ru^25^ and Ru^50^ have almost identical binding energies, Cu–Cu interactions and Cu–surface interactions, yet on the Ru^50^ surface the formation of an S + 2 layer is prevented even at 800 K. To understand what limits Cu migration and island formation, we study the specific activation energies for the migration of an atom between the various layers.

### Study of the growth mechanism of the Cu thin film

Studying the migration pathways of atoms into the S + 1 and S + 2 layers allows us to gain insight into the growth mechanism of a Cu structure on Ru-modified TaN and how the morphology of copper can be controlled by the Ru content in the TaN surface. Pathways were studied for the bare surface, Ru^25^ surface and Ru^50^ surface. The Ru^25^ surface allows us to explore the activation energies of a composition that appeared to promote formation of a third layer of copper. The Ru^100^ surface was not studied due to concerns relating to the electronic properties, morphology and thermal stability of the Cu structure with incorporated Ru and N.

For these calculations, we take the snapshot at the end of the 500 K MD simulation and relax them using the computational setup described in Section 2. We then allow one Cu atom to migrate from a layer to the next layer up (as defined in Fig. 2(c)). Activation barriers are presented in Table 2.

**Table 2:** Activation energies for NEB transitions on bare TaN, Ru^25^ and Ru^50^. Forward and reverse signify a Cu atom transition up one layer and down one layer, respectively. Migration pathways e1, e2, and e3 are defined in Fig. 2(c)

	e1		e2		e3	
	Forward (eV)	Reverse (eV)	Forward (eV)	Reverse (eV)	Forward (eV)	Reverse (eV)
TaN	0.84	0.44	0.76	0.74	0.70	0.33
Ru^25^	0.94	0.30	1.21	1.25	0.92	1.58
Ru^50^	1.18	1.08	1.86	1.82	0.62	0.78

On bare TaN, the activation barrier to Cu migration is 0.70–0.84 eV depending on the transition in question. The largest barrier is for Cu migration from surface bound Cu in the surface layer to the S + 1 layer. When we consider Cu migration to the S + 2 layer given the previous migration of copper, then the barrier is reduced. This is consistent with the idea that prior migration of a metal atom from the surface to the next layer can promote the migration of surface bound metal atoms.^41^ In particular, the e2 migration, where a Cu atom migrates up two layers (from the surface to the S + 2 layer) has a moderate activation barrier, suggesting that this transition will be important in 3D copper formation. The reverse migrations, *i.e.* where Cu migrates towards the surface, have lower activation barriers, suggesting that the process of 3D island formation will be dynamic.

**Fig. 6:**
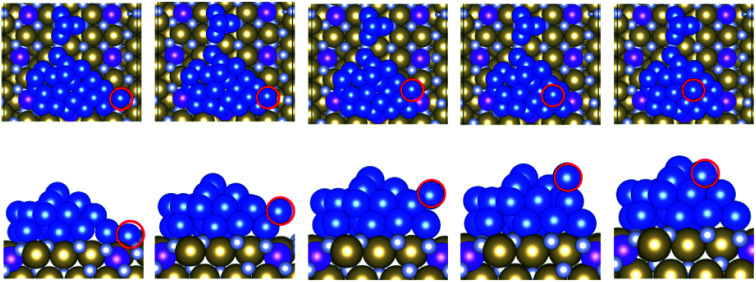
Transition of a copper atom from the surface layer to the S + 2 layer (e2) on Ru^25^. The top row shows the top view and the bottom row shows the side view. The migrating Cu atom is highlighted with a red outline.

When we consider the Ru-doped TaN systems, our results show that as little as 25% Ru doping increases the activation barrier to upward migration compared to bare TaN. For example, the e1 migration has a slightly increased activation barrier compared to TaN, while the reverse migration of copper has a decreased activation barrier indicating that it is difficult to promote the initial Cu migration to the S + 1 and S + 2 layers. This matches our previous understanding of Ru dopants acting as nucleation sites in the surface.^39^ The transition from the surface to the S + 2 layer (e2 in Fig. 2(c)) shows a strong increase in the activation energy compared to the bare surface. This is caused by the incorporation of the migrating atom into one of the surface gaps at the start of the migration, which is shown in Fig. 6 and is an important mechanism for the stabilisation of 2D copper on Ru-modified TaN.

Calculating the activation energy towards migration of a Cu atom from the S + 1 layer to the S + 2 layer (e1 in Fig. 2) on Ru^50^ shows that the presence of Ru significantly increases the energy required for this initial upward migration, from 0.84 eV on bare TaN to 1.18 eV. The activation energy for the reverse migration to the S + 1 layer is also increased compared to that of the bare TaN surface, to 1.08 eV. Similarly, both upward and reverse migrations from the surface layer to the S + 2 layer (e2 in Fig. 2(c)) exhibit increased activation barriers compared to bare TaN and Ru^25^. The transition of a second atom following the first S + 1 to S + 2 migration (e3 in Fig. 2(c)) has a decreased activation energy compared to bare TaN and Ru^25^ indicating that once the first barrier to migration has been overcome, upward migration is promoted. However, given that the activation energies for the initial upward migration are quite large, it is unlikely that this copper migration will take place at typical processing temperatures. Further, the literature indicates that as long as the metal–substrate interaction is strong enough, any upward migration becomes extremely difficult.^44^ This fits our study of Cu_2_ to Cu_4_ clusters on Ru-modified TaN.^9^ Here, we observed much lower metal–metal interaction energies overall compared to Cu_29_, but a stronger metal–metal interaction for surfaces with a high Ru content, and weaker metal–metal interactions for Ru^25^ and Ru^50^. However, both of the high Ru content surfaces showed extremely strong metal–substrate interactions with small Cu clusters, allowing us to conclude correctly that 2D growth would be promoted regardless. This trend is partially reversed here, with slightly weaker metal–metal interactions on surfaces with a high Ru content, while the interaction on Ru^25^ and Ru^50^ is very close to what is observed for bare TaN. This is likely due to a large contribution to the metal–metal interaction from atoms in the S + 1 and S + 2 layers.

**Fig. 7:**
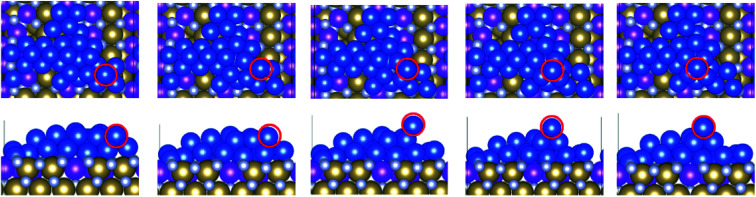
Transition of an atom from the S + 1 layer to the S + 2 layer (e1) on Ru^50^. The top row shows the top view and the bottom row the side view. The migrating Cu atom is highlighted with a red outline.

**Fig. 8:**
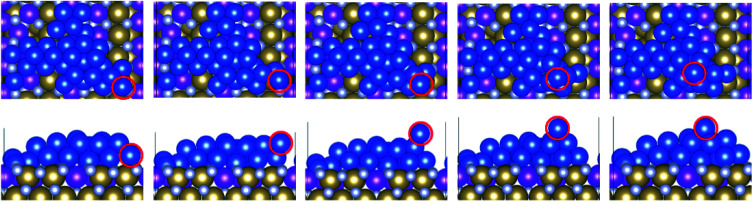
Transition of an atom from the surface layer to the S + 2 layer (e2) on Ru^50^. The top row shows the top view and the bottom row shows the side view. The migrating Cu atom is highlighted with a red outline.

Detailed atom migration for the e1 and e2 migrations on the Ru^50^ surface is shown in Fig. 7 and 8, respectively.

Overall, from the analysis of these Ru-TaN structures, our results indicate that if we want to incorporate Ru into TaN, then some Ta atoms are needed in the surface layer in order to ensure the thermal stability of the Ru-containing surface layer and reduce Cu, Ru and N migration around the interface region.

Based on our analysis, a Ru dopant content of around 50% appears to be suitable to promote 2D deposition of copper rather than 3D island growth. We can clearly see that as the Ru content increases, the Cu–substrate interaction also increases. However due to possible thermal instabilities, dopant concentrations above 50% are inadvisable, while at 50% doping the activation barrier for upward migration of copper to make the third and subsequent layers of the 3D structure is significantly increased when compared to bare TaN. Further, both Ru^25^ and Ru^50^ surfaces show Cu binding energies more favourable than the cohesive energy of Cu and no transition beyond a two-layer structure is observed with the increased temperature for Ru^50^ surfaces.

While the exact distribution of dopants in the surface cannot be controlled during the growth process, if the growth is driven by thermodynamics a structure like the one seen in the Ru^50-10^ would be obtained. Other 50% distributions have also shown promising results (see *T* = 0 K and 500 K MD results in Fig. S1 and S2 in the ESI†) so that the exact distribution should not affect the Cu growth to the point where island formation is promoted over a 2D morphology.

## 4. Conclusion

The search for new barrier + liner materials that will allow the continued use of copper in downscaling of CMOS devices is a significant challenge and one of the bottlenecks for further device scaling. We propose that the previously separate barrier and liner materials can be integrated into a single material, based on modifying the existing TaN barrier through surface doping, replacing Ta with Ru. A combination of static geometry relaxations, *ab initio* molecular dynamics at finite temperatures and the activation barrier from nudged-elastic band calculations of copper atom migrations, have demonstrated how the Ru content of Ru-modified TaN controls the 2D *versus* 3D morphology of the Cu_29_ structure, representative of a growing Cu thin-film. This Ru-TaN film can be deposited through atomic layer deposition which provides a high degree of control over the thickness and composition.

We show that the incorporation of Ru into the TaN surface strengthens the metal–substrate interaction and thus should inhibit the 3D growth of Cu on the surface. A dopant concentration of 50% in the surface layer is sufficient to promote 2D growth while also showing improved thermal stability compared to higher concentrations of Ru which show mixing of Cu, Ru and N at the interface. The 50% Ru doping increases the activation barrier to copper atom migration to upper layers and will therefore promote 2D growth of copper. This will result in deposition of conducting films rather than non-conducting islands. These findings further confirm our earlier hypotheses on the effect of Ru dopants in TaN on Cu growth based on our previous work on smaller Cu structures.

This new information gives vital insight into a solution for extending the use of Cu in interconnects, and a more detailed understanding of factors controlling 2D *vs.* 3D growth of metals on nitride substrates.

## Data availability

All necessary VASP5 input and output files for the calculations presented in this paper are available at the following repository: https://github.com/MMD-group/VASP/tree/master/Control%20of%20Cu%20morphology%20on%20Ru-passivated%20and%20Ru-doped%20TaN%20Surfaces%20%E2%80%93%20promoting%20growth%20of%202D%20conducting%20copper%20for%20CMOS%20interconnects.

## Author contributions

Authors CLN, SKN and MN together conceptualised the content of the work and paper, deciding on the calculations that were required. Authors CLN and SKN collaborated on the design of the models. Author CLN was in charge of running the calculations, selecting appropriate atom migration pathways for the different surface structures and carrying out all necessary analysis. Author CLN wrote the manuscript with detailed feedback and corrections from authors SKN and MN.

## Conflicts of interest

The authors have no conflicts of interest to report.

## Supplementary Material

SC-013-D1SC04708F-s001

SC-013-D1SC04708F-s002
