# Supplementary material for: Control of the Cu morphology on Ru-passivated and Ru-doped TaN surfaces – promoting growth of 2D conducting copper for CMOS interconnects
Source: Chem Sci. 2021 Dec 13;13(3):713–25. doi: 10.1039/d1sc04708f (PMC8768880; doi:10.1039/d1sc04708f)
Supplement: SC-013-D1SC04708F-s001 [file SC-013-D1SC04708F-s001.pdf]

Electronic Supplementary Material (ESI) for Chemical Science.
This journal is © The Royal Society of Chemistry 2021

1. Movie files are uploaded to the submission system. Text added on page 7: *Movie files (animated gifs) of AIMD runs at 500 K for TaN, 1ML-Ru on TaN, 50% Ru-TaN and 100% Ru-TaN have been provided.*
    a. These movies are available at this link: https://drive.google.com/drive/folders/12qOVV1KSPBgKNUcdg-eq64iA9LDmMYhR?usp=sharing
