# Supplementary material for: Control of the Cu morphology on Ru-passivated and Ru-doped TaN surfaces – promoting growth of 2D conducting copper for CMOS interconnects
Source: Chem Sci. 2021 Dec 13;13(3):713–25. doi: 10.1039/d1sc04708f (PMC8768880; doi:10.1039/d1sc04708f)
Supplement: SC-013-D1SC04708F-s002 [file SC-013-D1SC04708F-s002.pdf]

Electronic Supplementary Material (ESI) for Chemical Science.
This journal is © The Royal Society of Chemistry 2021

Supporting Information

Control of Cu morphology on Ru-passivated and Ru-doped TaN Surfaces – promoting growth of 2D conducting copper for CMOS interconnects

Cara-Lena Nies[1], Suresh Kondati Natarajan[2] and Michael Nolan[1,3*]

1 - Tyndall National Institute, University College Cork, Lee Maltings, Dyke Parade, Cork, T12 R5CP, Ireland

2 - Synopsys Denmark ApS, Fruebjergvej 3, 2100 Copenhagen, Denmark

3 - NIBEC, School of Engineering, University of Ulster at Jordanstown BT37 0QB, United Kingdom

Description of Models:

All models in this study are based on ε-TaN in the Fe2P crystal structure.[1] The bulk geometry was optimised by relaxing the ionic positions, cell volume and cell shape yielding the following equilibrium lattice parameters which agree closely with experimental results: a = b = 5.23 Å, c = 2.92 Å, α = β = 90° γ = 120°. [2] These calculations were part of a previous study carried out by the authors. [3] Surface models of the low index surfaces (1 1 1), (1 0 0) and (1 1 0) were created from the optimised bulk structure using the Atomic Simulation Environment (ASE) package. [4] As part of the same study, we found that the (1 1 0) surface is the lowest energy surface for TaN and is used as the basis for all TaN and Ru-modified TaN models presented in this work.
To accommodate large Cu clusters we use a (2 x 4) supercell with the following dimensions: a = 18.11 Å, b = 23.36 Å, c = 30.48 Å; α = β = γ = 90°. This includes a vacuum region of 12 Å. Ru doping is carried out by replacing a selection of surface Ta atoms with Ru atoms. Dopants are added only in the top layer of the slab to ensure the preservation of the barrier properties of TaN. The doping process and a study of the effects of varying the distribution of dopants in the surface were published as part of a previous study carried out by the authors. [5] The minimum distance between Cu atoms in neighbouring images when adsorbing the original Cu29 structure is 6.41 Å.

Figures S1 and S2 show the 0K and 500K structures of Cu_{29} on two different Ru^{50} surfaces with varying Ru distribution. Ru^{50-1} has Ru dopants only at site S (6 coordinate) and Ru^{50-4} has dopants at both site S and F (3 coordinate).

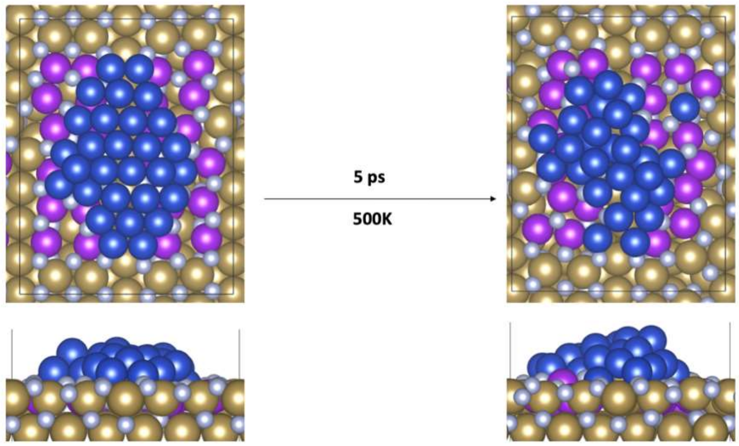

Figure S1: Structures of Cu_{29} obtained after relaxation and 500 K MD on $Ru^{50\text{-}1}$

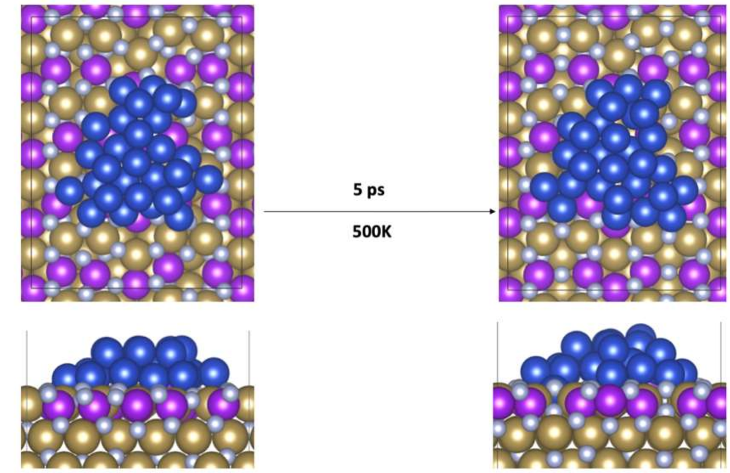

Figure S2: Structures of Cu_{29} obtained after relaxation and 500 K MD on $Ru^{50\text{-}4}$

Geometries after 5 ps of MD at 300 K, 500 K and 800 K for all surfaces are shown in Figures S3.

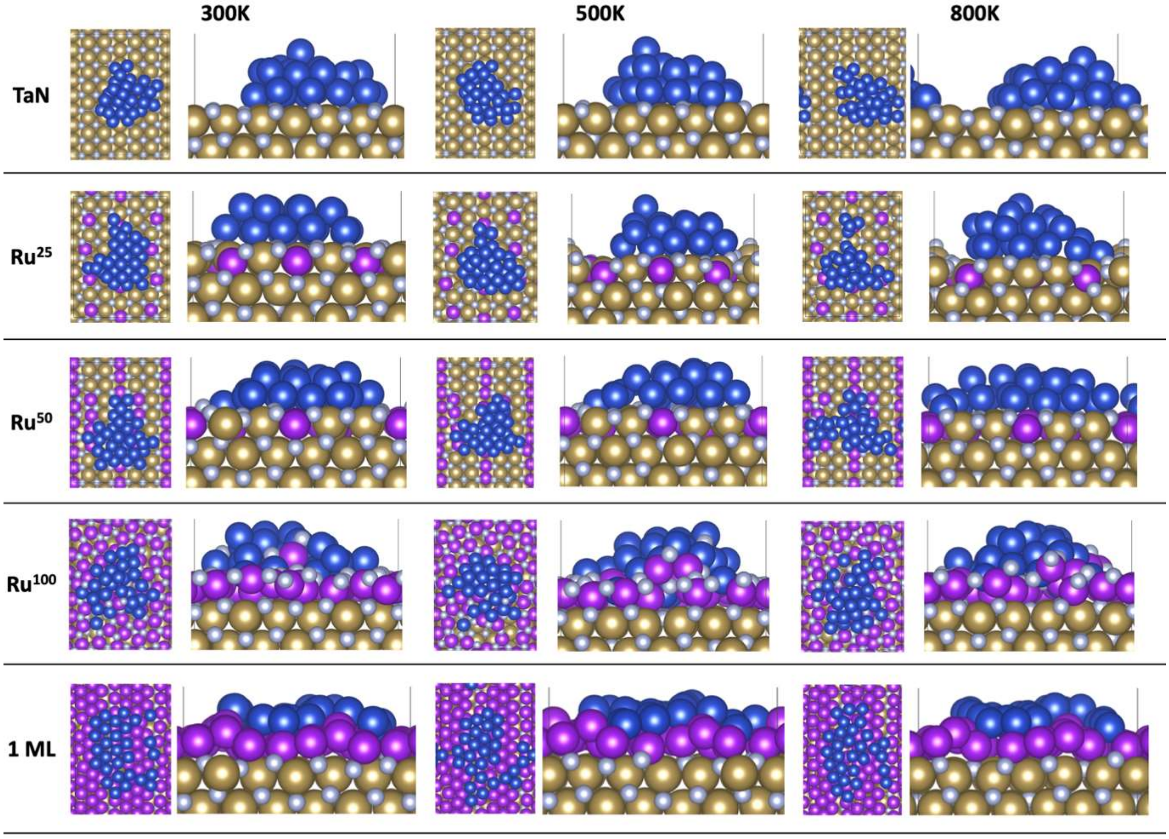

Figure S3: Geometries obtained after 300 K, 500 K and 800 K MD runs for TaN, Ru^{25}, Ru^{50}, Ru^{100} and 1ML passivated TaN.

Changes in z-position during AIMD calculations are shown in the Figures S4, S5, S7 and S8 below. Figure S6 shows the changes in z-position during the geometry relaxation of Cu_{29} on Ru^{100}.

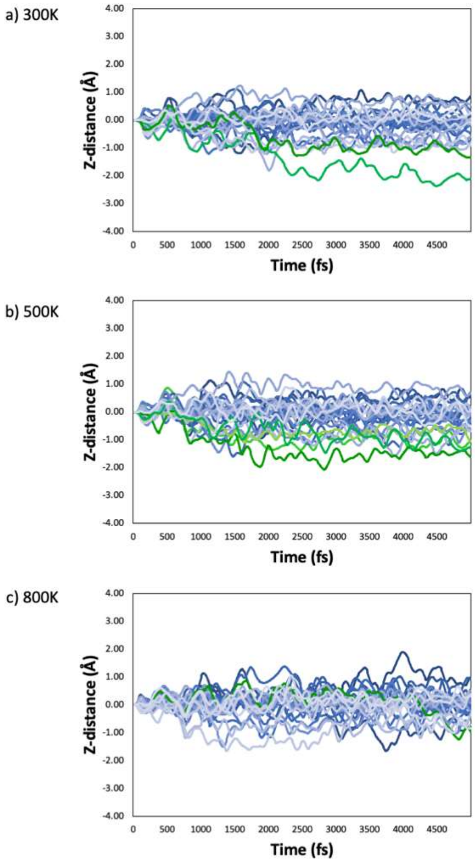

Figure S4: Change in Z-position with respect to time for Cu29 on 1ML Ru-passivated TaN during an MD run at 3 different temperatures.

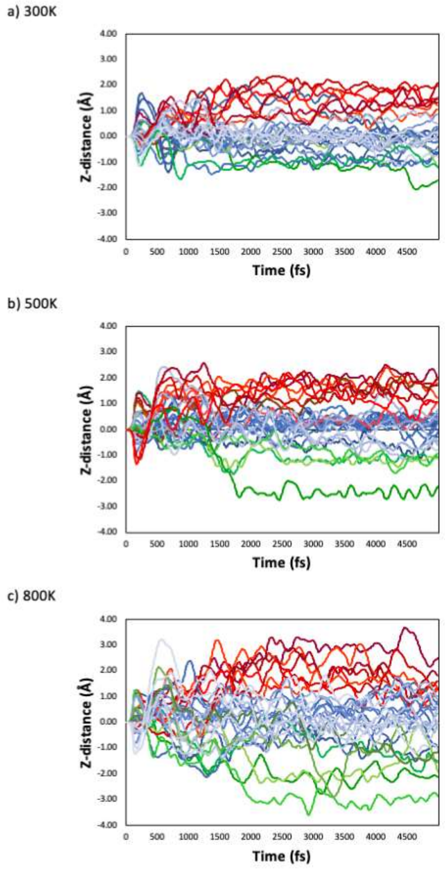

Figure S5: Change in Z-position with respect to time for Cu29 on Ru[100] during an MD run at 3 different temperatures.

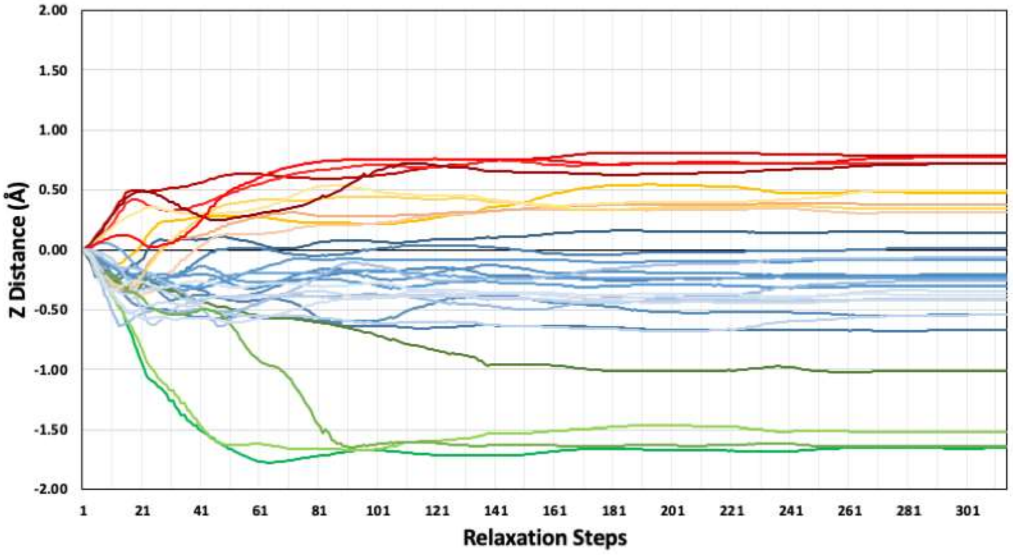

Figure S6: Change of Z position with respect to relaxations steps for Cu_{29} on Ru^{100}. Each line represents one Cu atom. Blue lines are atoms that did not move from their original Z position. Atoms in yellow are slightly elevated but are not part of the second layer. Red lines indicate atoms in the second layer, while green lines are for atoms that have migrated into the top surface layer.

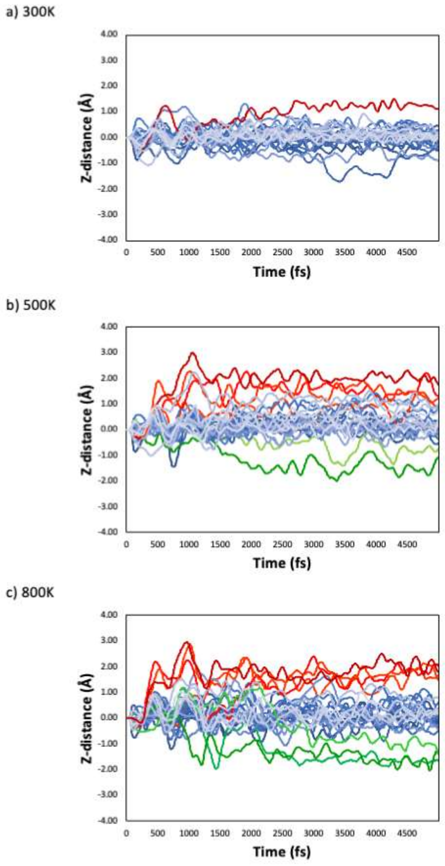

Figure S7: Change in Z-position with respect to time for Cu29 on Ru[50] during an MD run at 3 different temperatures.

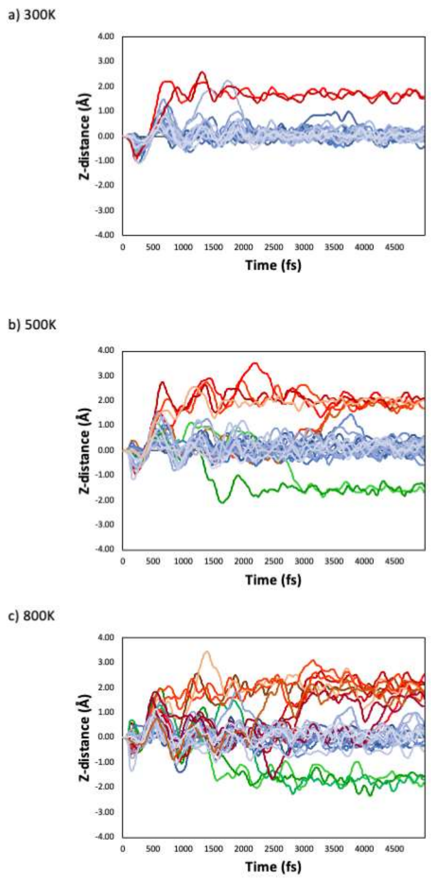

Figure S8: Change in Z-position with respect to time for Cu29 on Ru[25] during an MD run at 3 different temperatures.

Discussion of z-plots Ru[100]

The Cu structure remains relatively flat during the geometry relaxation, as illustrated by the z-plots in Figure S6. However, during the MD calculations, Cu atoms not only migrate into the surface layer, but Ru and N atoms also migrate out of the surface, which leads to a mixture of Cu, Ru and N instead of a clean Cu_{29} structure. Further, the atoms begin to associate and while there are no distinct layers as observed on other surfaces, the mixture is no longer a single layer film. This is illustrated further through the plots in Figure S5. Here, there are many atoms that have migrated to ± 1Å from their original positions. Further, in Figure S3, we can see the rise of Ru and N atoms out of the Ru-TaN structure, which is not reflected in the z-plot. However, a potential replacement of Ru and N atoms with Cu atoms could account for the Cu atoms that are displaced to nearly -3 Å at 800K.

Discussion of z-plots Ru^{50}

Similarly to Cu_{29} adsorption on the passivated surface, at 800 K the atoms begin to interact at the periodic boundary, forming the beginning of a pseudo 1D ribbon structure. Some migration into surface recesses is observed, but no third layer is formed. Figure S7 shows how the displacement of atoms along the Z axis changes with increasing temperature. The larger number of atoms in the central area (coloured in blue) shows that the majority of atoms do not move away from their original location. This confirms that the structure remains as a two-layer system. As temperature increases, there are several atoms displaced by around 1.5 Å from their origin, while 2 and 3 atoms migrate into a recess and are displaced by around -2.0 Å.

Discussion of z-plots Ru^{25}

At 800 K, there are strong distortions to the structure of copper and a tetrahedral Cu_4 appears to split off from the remainder of the structure, which remains as a two-layer structure with two copper atoms having migrated upwards into the *S+2* layer and 3 atoms burying into one of the recesses created by the surface Ru. This is visible in the z-plots (see Figure S8), although it is difficult to distinguish *S+2* layer atoms from *S+1* layer atoms. These are best identified visually (see Figure S3).

DOS plots and Bader Charges

In general, atoms bound to the surface are more strongly oxidized than what was observed for any of the other surfaces, with computed charges ranging from 10.5 to 10.9 electrons. Cu atoms solely bound to other Cu atoms once again remain metallic.
Those atoms that are slightly elevated from the surface layer, but not fully in a *S+1* layer remain metallic. Atoms incorporated into the surface layer are found to be more oxidized than atoms on the surface, with computed charges of 10.4 to 10.6 electrons.

DOS plots, Figure S9, and Bader charge analysis show that 1 ML Ru-modified TaN is metallic, and copper also remains metallic. Copper atoms that are directly bound to the surface are partially oxidised, with computed Bader charges between 10.6 and 10.9 electrons. Atoms in the second layer of the cluster are metallic, with computed Bader charges of 11.0 electrons. For the 1ML passivated surface Bader analysis shows that Cu atoms remain metallic. This indicates that there is no charge transfer between the Cu adatoms and the Ru atoms in the passivation layer.

The DOS of Ru^{25} and Ru^{50}, as shown in Figure S9, shows a very weak contribution from the Cu d orbitals, in contrast to the much stronger contribution from the Ru d orbitals. This is unexpected, as there are less Ru atoms in the system than Cu atoms and as the number of Ru atoms is doubled for Ru^{50}. Similarly, to the bare surface, the atoms bound to the surface are slightly oxidised with computed Bader charges of around 10.8 electrons, while atoms in the second layer have remain fully metallic.

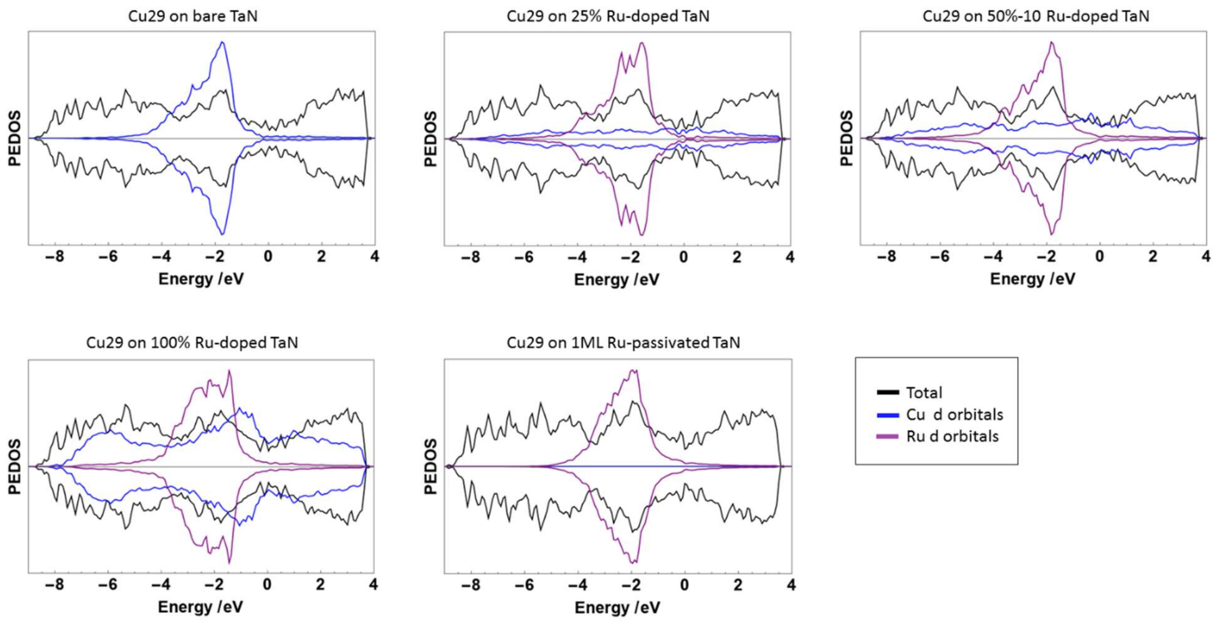

Figure S9: Density of State plots for all Cu_{29} adsorptions. The Cu and Ru contributions have been multiplied by 5 for easier comparison.

Possible Effect of Thermal Expansion

To explore if there is any potential impact as a result of thermal expansion of TaN and RuTaN at the AIMD temperatures, we carried out NPT ensemble AIMD calculations of (3x3x3) supercells of bulk TaN and 11% Ru doped TaN. The latter was achieved by replacing one in every three Ta atoms in one layer of the bulk with Ru (as a model system). The AIMD calculations were run at a temperature of 800 K with 1 bar of pressure for up to 2 ps. The original lattice parameters (based on the experimental crystal structure of TaN available in the Materials Project Database at https://materialsproject.org/materials/mp-1279/), were: a = b = 5.23 Å, c = 2.93 Å, α = β = 90°, γ = 120°. Throughout the AIMD run the lattice parameters for Ru-doped TaN varied from the original as follows: a = –0.38 % to –3.60 %, b = –0.74 % to –3.38 %, c = +0.34 % to – 5.58 %; please note that the a and b parameters contract and do not show any expansion. The corresponding changes in lattice vectors for undoped TaN are quite similar: a = –0.76 % to –3.70 %, b = – 0.52 % to –3.38 %, c = +0.34 % to –5.58 %. From the geometry we observe that as on the Ru-doped TaN surfaces, small cavities form around the dopants due to the difference in ionic radius of the two metals. However, the overall decrease in lattice parameters indicates that Ru-doped TaN should have a negative thermal expansion coefficient and shrink at higher temperatures, which rules out the possibility of thermal

expansion causing further cavities that impact the diffusion barrier properties of the material.

References:

[1] K. Persson, “Materials Data on TaN by Materials Project.” 2015, doi: 10.17188/1189196.

[2] A. Christensen and B. Lebech, “Reinvestigation of structure of epsilon-tantalum nitride,” *Acta Crystallogr. B*, vol. 34, no. JAN, pp. 261–263, 1978, doi: 10.1107/S0567740878002733.

[3] S. Kondati Natarajan, C.-L. Nies, and M. Nolan, “Ru passivated and Ru doped ε-TaN surfaces as a combined barrier and liner material for copper interconnects: a first principles study,” *J. Mater. Chem. C*, vol. 7, no. 26, pp. 7959–7973, Jul. 2019, doi: 10.1039/C8TC06118A.

[4] A. Hjorth Larsen *et al.*, “The atomic simulation environment-a Python library for working with atoms,” *J. Phys. Condens. Matter*, vol. 29, no. 27, pp. 273002–273030, Jul. 2017, doi: 10.1088/1361-648X/aa680e.

[5] S. Kondati Natarajan, C.-L. Nies, and M. Nolan, “The role of Ru passivation and doping on the barrier and seed layer properties of Ru-modified TaN for copper interconnects,” *J. Chem. Phys.*, vol. 152, no. 14, p. 144701, Apr. 2020, doi: 10.1063/5.0003852.
